# Effects of covid-19 infection on thyroid functions

**DOI:** 10.5937/jomb0-34934

**Published:** 2022-10-15

**Authors:** Deniz Gezer, Müzeyyen Seval Ecin

**Affiliations:** 1 Mersin City Training and Research Hospital, Unit of Internal Medicine Clinic, Mersin, Turkey; 2 Mersin City Training and Research Hospital, Unit of Occupational Diseases Clinic, Mersin, Turkey

**Keywords:** Covid-19, Sars-CoV-2, thyroid gland, thyroid function tests, euthyroid sick syndrome, Covid-19, Sars-CoV-2, štitna žlezda, testovi funkcije štitne žlezde, sindrom bolesti eutireoidne žlezde

## Abstract

**Background:**

COVID-19 may affect many endocrine tissues as well as thyroid gland and hypothalamus-pituitary-thyroid axis. It has been shown that COV D-19 infection suppresses thyroid hormones in some studies and causes subacute thyroiditis in the others so that its effects are still not fully known. The aim of our study is to retrospectively evaluate thyroid functions, clinical findings, biochemical and inflammatory markers in PCR positive patients infected with COVID-19; and to evaluate the relationship between abnormal thyroid function tests (TFT) and clinical and laboratory findings and whether it has potential prognostic significance.

**Methods:**

The data of patients aged 18 years and older, 201 patients who applied to Mersin City Training and Research Hospital due to COVID-19 infection between 1st of March and 1st of April in 2021 and received inpatient treatment were evaluated retrospectively

**Results:**

Large TFT (TSH, T3, T4, anti-TPO) and laboratory data of 201 patients with mild, moderate or severe pneumonia on CT were scanned retrospectively. 121 (60.2%) of the patients were male, mean age was 51.9 ± 14.6 years, and the most common comorbid disease was hypertension in 65 (32.3%) patients.

**Conclusions:**

It has been determined that the deterioration in TFTs is associated with LDH and D-dimer which are indicators of cell and endothelial damage, duration of hospitalization, clinical severity, and having mutant strains and it has been concluded that low TSH can be used as a prognostic indicator in COVID-19 patients. Further studies with healthy control groups, quantitative RT-PCR tests, histological and pathological correlations, and long-term follow-up are needed.

## Abstract (sr)

**Uvod:**

COVID-19 može uticati na mnoga endokrina tkiva, kao i na štitnu žlezdu i osovinu hipotalamus-hipofiza-štitna žlezda. Pokazalo se da infekcija COVID-19 potiskuje hormone štitne žlezde u nekim studijama, a izaziva subakutni tiroiditis u drugim, tako da njeni efekti još uvek nisu u potpunosti poznati. Cilj našeg istraživanja je da se retrospektivno proceni funkcije štitne žlezde, klinički nalazi, biohemijski i inflamatorni markeri kod PCR pozitivnih pacijenata inficiranih COVID-19; i da proceni odnos između abnormalnih testova funkcije štitaste žlezde (TFT) i kliničkih i laboratorijskih nalaza i da li ima potencijalni prognostički značaj.

**Metode:**

Podaci o 201 pacijenta starijih od 18 godina, koji su se prijavili u bolnicu za obuku i istraživanje u gradu Mersin zbog COVID-19 infekcije između 1. marta i 1. aprila 2021. godine i koji su primili bolničko lečenje, procenjeni su retrospektivno.

**Rezultati:**

Retrospektivno su skenirani veliki TFT (TSH, T3, T4, anti-TPO) i laboratorijski podaci 201 pacijenta sa blagom, umerenom ili teškom pneumonijom na CT. 121 (60,2%) obolelih bilo je mu{kog pola, prosečne starosti 51,9 ± 14,6 godina, a načešća komorbidna bolest bila je hipertenzija kod 65 (32,3%) bolesnika.

**Zaključak:**

Utvrđeno je da je pogoršanje TFT-a povezano sa LDH i D-dimerom koji su indikatori oštećenja ćelija i endo-tela, trajanja hospitalizacije, kliničke težine i postojanja mutantnih sojeva i zaključeno je da se može koristiti nizak TSH kao prognostički indikator kod pacijenata sa COVID-19. Potrebne su dalje studije sa zdravim kontrolnim grupama, kvantitativni RT-PCR testovi, histološke i patološke korelacije i dugotrajno praćenje.

## Introduction

COVID-19 infection, a corona virus associated with SARS-CoV-2, was first reported from China towards the end of 2019 and was recognized as a pandemic by the World Health Organization (WHO) in March 2020 [1]. COVID-19 affects many endocrine tissues as well as thyroid tissues. In postmortem studies, it has been shown to affect follicular and parafollicular cells in the thyroid gland and the pituitary gland [2] [3]. In addition, SARS-CoV-2 enters cells by using angiotensin converting enzyme which is highly expressed in the thyroid gland [4]. Therefore, the hypothalamus-pituitary-thyroid axis may be affected in patients with COVID-19. However, since it suppresses thyroid hormones in some studies and causes subacute thyroiditis in others so that its effects are still not fully known [5] [6] [7] [8].

Therefore, in this study, we aimed to evaluate thyroid functions, clinical findings, biochemical and inflammatory markers in patients with COVID-19; the associated with abnormal thyroid function tests (TFT) and clinical/laboratory findings, and their potential prognostic significance.

## Materials and methods

The data of patients aged 18 years and older who applied to Mersin City Training and Research Hospital due to COVID-19 infection between 1st of March and 1st of April in 2021 and received inpatient treatment were evaluated retrospectively. Tests taken within the first 48 hours of hospitalization were included in the study. Thyroid stimulating hormone (TSH), free triiodothyronine (fT3) and free thyroxine (fT4) with Anti Thyroglobin (Anti-TPO) chemiluminescence immunoassay (Siemens Healthcare Diagno stics Inc, Laboratory Diagnostics, Advia Centaur XPT, Erlangen, Germany, produced in Ireland) were analyzed. Reference range of serum TSH concentration is 0.4 to 4.2 mU/L, fT4 9 to 30 pmol/L (0.7-2.5 ng/dL), fT3 3.5 to 6.5 pmol/L (0.22-0.43 ng/dL) as reference range received [9] [10]. fT3/fT4 <0.3 was accepted as a risk for thyroiditis [11].

Hematological (hemoglobin [Hb], white blood cell [WBC) and biochemical parameters (creatinine, aspartate transaminase [AST], alanine aminotransferase [ALT], lactate dehydrogenase [LDH]), in flammatory markers [C-reactive protein (CRP), pro-calcitonin], ferritin], thorax computed tomography (Thorax CT) (CT) scan (GE Healthcare Optima CT660, Chicago, IL, USA) were scanned retrospectively. Presence of SARS-CoV-2 reverse trans cription-polymerase chain reaction (RT-PCR) from nasopharyngeal swab and/or deep throat saliva Bio-Speedy COVID-19 RT-qPCR kit BS-SY-WCOR-305-1000; Patients who tested positive using the version 2003261000SK-MK kit were included. Demographic data and comorbid diseases, oxygen saturation (SpO_2_) obtained by pulse oximetry, respiratory rate, fever, need for intensive care, and which treatments were obtained by retrospective system scan. Patients with previous thyroid disease took antithyroid drugs or thyroid replacement therapy, amiodarone and heparin therapy that impair thyroid function or influence TFTs were not included in the study. The severity of COVID-19 disease was defined according to the »Chinese Clinical COVID-19 Pneumonia Diagnosis and Treatment (7th edition)« guideline. Mild clinical condition but normal imaging as mild disease; fever and respiratory symptoms with imaging findings as moderate disease; respiratory rate 30/min, SpO_2_ at rest ≤ 93%, and imaging >50% increase within 48 hours and having more than one of these conditions were defined as severe disease [12].

Data were analyzed using the statistical program SPSS 21.0 (IBM Corp., Armonk, NY, USA). For data not normally distributed, as determined by the Kolmogorov-Smirnov. Median ± SD (standard deviation) was used to define numerical variables, and categorical variables were defined as numbers and percentages. Between-group comparisons were performed with the t test and the Mann-Whitney U test for continuous variables, and the chi-squared or Fisher exact tests for categorical variables as appropriate. Multivariable logistic regression was used to show the relationship between patients with low TSH levels and other independent variables. The study protocol was approved by the non-interventional clinical research ethics board of the Mersin University. (2021/456)

## Results

Our study included 201 patients who applied to Mersin City Training and Research Hospital between 1st of March and 1st of April 2021 due to COVID-19 infection and were hospitalized. 121 (60.2%) were male, with a mean age of 51.9 ± 14.6 years. All our patients are PCR + and do not have any previous thyroid diseases. In 65 (32.3%) patients, the most common comorbidity disease was hypertension.

185 (91.5%) of the patients had symptoms and the most common symptoms were dispone and back pain. Clinical 142 (70.6%) of the patients were moderate, CT findings were 129 (64.2%) severe, and the mean length of stay in hospital was 9.9 ± 5.8. Steroid therapy was started in all hospitalized patients. 110 (54.7%) low TSH ((35 (17.4%) patients had subclinical hyperthyroidism), 116 (57.7%) low T3, 42(20.9%) low T4, 3(1.5%) high T4, 33(16.4%) anti-TPO elevation was detected. Isolated low TSH was detected in 5 (2.5%) subclinical hyperthyroidisms due to autoimmunity was found. Of 116 patients with low T3, 68 (33.8%) had concomitant low TSH, 33 (16.4%) had isolated low T3, and 12 (5.9%) had low T3 and T4 were determined. In our study, 35 (17.4%) patients had isolated low TSH, and 4 (2%) patients had elevated TSH, and 3 (1.5%) of these patients were accompanied by low T3 and T4, and Anti-TPO was negative. Demographic and laboratory data of the patients are given in Table 1.

**Table 1:** Demographic and laboratory data of the patients. Data mean ± SD, median (min-max), number (%) as appropriate.<br>Abbreviations: PCR: polymerase chain reaction, TSH: thyroid stimulating hormone, T3: triiodothyronine, T4: thyroxine, TPO: Thyroglobin, Hb: Hemoglobin, WBC: White blood cell, AST: Aspartate transaminase, ALT: Alanine aminotransferase, LDH: lactate dehydrogenase, CRP :C-reactive protein, ESR: Erythrocyte sedimentation rate

Features	N :201
Gender	
Female	80 (30.8)
Male	121(60.2)
Age mean±SD	51.9 ± 14.6
Comorbidity	
Cardiovascular disease	25(12.4)
Hypertension	65 (32.3)
Respiratory diseases	26 (12.9)
Steroid usage	201(100)
Symptoms	
Fever	98 (48.8)
Sore throat	45 (22.4)
Back pain	104(51.7)
Dispne	184(91.5)
Taste-smell loss	2(1)
Oxygen requirement	177(17.7)
CT	
Mild	44(21.9)
Modarete	129 (64.2)
Severe	28 (13.9)
Clinic	
Moderate	142 (70.6)
Severe	59 (29.4)
Plasmapheresis	3 (1.5)
Cytokine Filter	1(1)
Intensive Care	44(21.9)
ntubated	10(5)
Death	11(5.5)
Length of Stay in Hospital	9.9±5.8
TSH, mIU/L	0.8±1.4
Low	110(54.7)
Normal	87(43.3)
High	4(2)
T3, pmol/L	2.3±0.5
Low	116(11.6)
Normal	85(8.5)
T4, pmol/L	1.1±0.2
Low	42(20.9)
Normal	156(15.6)
High	3(1.5)
TPO, kU/L	
Normal	168(83.6)
High	33((16.4)
Mutant	
Yes	24 (11.9)
No	177 (88.1)
WBC, 10^9^x/L	6125(2400–17430)
HB, g/L	13.8(7.1–19.0)
Glucose, mmol/L	130(80–440)
Creatinine, μmol/L	0.8(0.3–5.1)
AST, U/L	32(12–399)
ALT, U/L	29(9–246)
LDH, U/L	291(163–670)
ESR, mm/h	22.5(2–94)
CRP, mg/L	4.33(0–35)
Procalcitonin, μmol/L	0.02 (0.01–1.86)
Ferritin, μg/L	252 (6–1771)
D-dimer, μg/L	0.55 (0.15–21.75)

In our study, increased length of hospital stays (p=0.04), clinical severity (p=0.05), high LDH level (p= 0.008) and high D-dimer (p=0.02) were found significant in patients with low TSH. TSH with moderate clinical severity levels were 0.7 (0.01-10.5) uIU/mL, TSH with severe severity levels were 0.9 (0.07-6.85) uIU/mL. Low TSH was detected more frequently in moderate patients. Comparison between patients with low TSH and normal TSH shown in Table 2.

**Table 2:** Comparison between patients with low TSH and normal TSH. Data mean ± SD, median (min-max), number (%) as appropriate.<br>Abbreviations: PCR: polymerase chain reaction, TSH: thyroid stimulating hormone, T3: triiodothyronine, T4: thyroxine, TPO: Thyroglobin, Hb: Hemoglobin, WBC: White blood cell, AST: Aspartate transaminase, ALT: Alanine aminotransferase, LDH: lactate dehydrogenase, CRP: C-reactive protein, ESR: Erythrocyte sedimentation rate<br>^**^2 patients with Brazilian mutation, others British mutation

	TSH low (N:110)	TSH Normal (N:91)	P value
Male	63 (57.3)	58(63.7)	0.4
Age	51.8±14.4	52.5±13.7	0.7
CT			
Mild	25(22.7)	19(20.9)	0.9
Modarete	69(62.7)	60 (65.9)	
Severe	16(14.5)	12 (13.2)	
Clinic			0.05
Modarete	84 (76.4)	58(63.7)	
Severe	26(23.6)	33 (36.3)	
Plasmapheresis	2(1.8)	1(1.1)	1
Intensive Care	21(19.1)	23(25.3)	0.3
Length of Stay<br>in Hospital	10.1±6.1	8.4±4.6	0.04
Mutant strain^*^	7 (6.4)	17 (18.7)	0.07
sT3, pmol/L	2.2±0.5	2.3±0.6	0.2
sT4, pmol/L	1.1±0.2	1.1±0.2	0.2
Anti TPO (+)	15(13.6)	18(19.8)	0.2
WBC, 10^9^x/L	5915(2780–17430)	6380(2400–17200)	0.1
HB, g/L	13.6±1.9	13.5±1.9	0.9
Glucose, mmol/L	126 (81–544)	129(80–440)	0.9
Creatinine mg/L	0.9(0.5–5.1)	0.9 (0.3–2.1)	0.5
AST, U/L	34(12–399)	30 (13–117)	0.06
ALT, U/L	31(9–246)	29 (10–198)	0.9
LDH, U/L	305(72-670)	276 (172–519)	0.008
ESR, mm/h	23(1–72)	23 (4–94)	0.9
CRP, mg/L	5.3 (0–16)	3.32 (0–35)	0.2
Procalcitonin,<br>μg/L	0.02(0.01–1.86)	0.01 (0.01–0.9)	0.3
Ferritin, μg/L	304(11–1771)	213 (5–3229)	0.2
D-dimer, μg/L	0.6(0.2–22)	0.5 (0.15–10.5)	0.02

In the multivariable logistic regression analysis, clinical severity (OR: 2.5, 95% CI: 0.07-1.2-5.6, p: 0.002) mutant strain (OR: 0.3, 95% CI: 0.1-0.8-0.6, p: 0.002) and high LDH (OR: 0.9, 95% CI: 0.9-1.0, p: 0.002) was found to be significant. The adjusted for age and gender in multivariable logistic regression analysis showing the relationship between low TSH and normal TSH is given in Table 3.

**Table 3:** The adjusted for age and gender in multivariable logistic regression analysis showing the relationship between low TSH and normal TSH. Significant P values are indicated in bold.<br>CT: Computed tomography, LDH: Lactate dehydrogenase.<br>^*^2 patients with Brazilian mutation, others British mutation

Features	OR (95% CI)	P-value
Age	1.0 (0.9–1.0)	0.9
Gender	0.8 (0.5–1.5)	0.6
Clinic	2.5 (1.2–5.6)	0.02
CT	0.4 (0.1–1.1)	0.08
Mutant strain^*^	0.3 (0.1–0.8)	0.02
Length of Stay in Hospital	0.9 (0.9–1.0)	0.3
LDH	0.9 (0.9–1.0)	0.02
D-dimer	0.9 (0.7–1.1)	0.3

## Discussion

In our study, large TFT (TSH, T3, T4, anti-TPO) and laboratory data of 201 patients with mild, moderate or severe pneumonia on CT who were PCR+ were scanned retrospectively. 121 (60.2%) of the patients were male, mean age was 51.9 ± 14.6 years, and the most common comorbid disease was hypertension 65 (32.3%). In the cohort studies, the mean age range was 49-56 years, and it was found to be more common in males. The most common comorbid disease was hypertension (30%), which is consistent with the literature [13] [14] [15].

In China Lui et al. [1] 13.1% abnormal thyroid dysfunction was detected in mild to moderate COVID-19 patients, Lania et al. [7] 25.4% and our study 57.7%. SARS-CoV-2 affects many cells both by using ACE receptors and via inflammatory cytokines, as well as disrupting TFT in this way. Hospitalization is planned for patients presenting with COVID-19 if they have CT involvement and/or SpO_2_ <93 in our hospital. The high rate of TFT disorder in our study is due to the fact that all of the patients had pneu monia and had moderate or severe clinical mani festations.

In a study with 50 patients COVID-19 in China, a decrease in TSH level was found in 56%, and our study 54.7% [8]. Other studies in the literature, TSH decrease varies between 5.2-15% [1] [6] [7]. 6 mg/day dexamethasone or 0.5-1 mg/kg/day methylprednisolone treatment was started for severe and critical COVID-19 patients and high-dose steroid (≥250 mg/day methyl prednisolone, 3 days) treatment was started in patients whose oxygen demand increased or acute phase reactants increased within 24 hours despite this treatment in our hospital with the guideline published by the Ministry of Health [16]. As it is known, steroid has a suppressive effect on TSH [17] and in our study like Chen et al. [8] exogenous steroid intake could not be excluded, so that our rates were high due to steroid treatment was started in all patients as a confounding factor effect.

191 patients with mild and moderate clinics in China, 4.7% patients had subclinical hyperthyroidism, 1% patients had subclinical hyperthyroidism due to autoimmunity, 10 patients isolated low T3 [1]. In our study 17.4% patients had subclinical hyperthyroidism, 2.5% subclinical hyperthyroidisms due to autoimmunity and 33 patients isolated low T3 was found. Since the clinical severity of the patients in our study was moderate and severe, it was concluded that as the clinical severity increased, cell damage increased as well as triggered autoimmunity. Even if the hypothalamus, pituitary and thyroid axis are not affected as the clinic gets worse, T3 decreases and rT3 increases, which is defined as euthyroidal illness syndrome (NTIS) [18].

Dosi et al. [19] found hypothyroidism in 2.5%. In our study, TSH elevation was found in 2%, which is consistent with the literature [20].

Clinical severity, increased length of hospital stays, elevated LDH elevation, which is one of the indicators of tissue damage, D-dimer, which is an indicator of endothelial damage were significant in patients with low TSH [20]. Low TSH levels were found to be higher in patients with moderate disease than those with severe disease.

It should be kept in mind that COVID-19 infection may impair TFT tests not only in severe disease, but also in moderate disease. It also causes tissue and endothelial damage due to systemic inflammation, prolonging the duration of hospitalization, and TFTs may also have prognostic value.

It has been concluded that having a mutant strain (Brazilian in 2 patients, British variant in other patients) may cause more TSH decrease, and TFT evaluation and follow-up is required, especially in mutant strains.

The absence of a healthy control group, the fact that methods such as ultrasonography and scintigraphy were not used to evaluate thyroiditis, the long-term follow-up of patients with disorders in TFT was not performed, steroids were started in all patients, and the quantitative RT PCR method was not used are the limitations of our study. To the best of our knowledge, being one of the first studies to determine the relationship between COVID-19 and TFT in Turkey, having been studied on a large patient population, and indicators of cell and endothelial damage such as LDH and D-dimer, length of hospital stay and clinical severity, and showing the relationship between low TSH in mutant strains and that low TSH can also be used as a prognostic indicator are the strengths of our article.

### Conclusion-Suggestions

COVID-19 may affect many endocrine tissues as well as thyroid glands and hypothalamus-pituitary-thyroid axis [2] [3]. It was concluded that de terio ration in TFTs is associated with indicators of cell and endothelial damage such as LDH and D-dimer, length of stay in hospital, clinical severity, having mutant strains, and low TSH can be used as a prognostic indicator. Further studies with healthy control groups, quantitative RT-PCR tests, having histological and patho logical correlations, and long-term follow-up are needed.

## Dodatak

### Acknowledgement

The authors report no conflicts of interest. The authors alone are responsible for the content and writing of the article.

### Author Contributions

Conception or design: D.G, S.M.E.,. analysis, or interpretation of data: S.M.E. Drafting the work or revising: D.G. Final approval of the manuscript: D.G., S.M.E.

### Conflict of interest statement

All the authors declare that they have no conflict of interest in this work.
